# Effects of an Astragalus Polysaccharide and Rhein Combination on Apoptosis in Rats with Chronic Renal Failure

**DOI:** 10.1155/2014/271862

**Published:** 2014-03-10

**Authors:** Yonghong Lian, Linlin Xie, Minghao Chen, Liguo Chen

**Affiliations:** ^1^Jinan University, Guangzhou 510632, China; ^2^Southern Medical University, Guangzhou 510515, China; ^3^Institute of Integrated Chinese and Western Medicine, Jinan University, Guangzhou 510632, China

## Abstract

*Objective.* To investigate the effects and to analyze the mechanism of the combination of Astragalus polysaccharide (APS) and Rhein on apoptosis in rats with chronic renal failure (CRF). *Methods.* Thirty-seven male Wistar rats were randomly divided into a control group, a model group, a low-dose APS and Rhein combination group, and a high-dose APS and Rhein combination group. CRF was induced by orogastric gavage with adenine. Rats were observed for renal function, electrolyte, and pathological changes for 7 weeks after administration. Renal tubular cell apoptosis was assessed by TUNEL and protein expressions of IRE1 and CHOP were detected by Western-blotting. *Results.* The combination of APS and Rhein decreased the kidney weight and index, improved renal pathological injury, maintained the stability of serum electrolytes, and reduced SCr and BUN levels in rat models. Moreover, APS and Rhein combination could effectively inhibit the apoptosis and reduce the protein expressions of IRE1and CHOP of renal tubular cells. *Conclusions.* The combination of APS and Rhein could improve renal function and reduce renal cell apoptosis to protect against further progression of CRF, whose mechanism may be related to alleviate endoplasmic reticulum stress (ERS) in the renal cells.

## 1. Introduction

As the number of patients worldwide with chronic renal failure (CRF) increases at an alarming rate, CRF has become a major public health problem. Compared to patients with other diseases, patients with CRF require more frequent and longer hospitalizations and have a poorer quality of life and a higher morbidity and mortality. Therefore, it is imperative to develop new medicines and alternative therapies for CRF [1]. In China, CRF is treated by means of Western medicine combined with traditional Chinese medicine (TCM). Therefore, effectiveness and feasibility of preventing and treating CRF have become one of the key points in the field of renal diseases. TCM herbs given orally or rectally are an economical treatment for CRF with Chinese characteristics. Related experimental studies on the clinical efficacy of TCM herbs have been widely reported. Of these studies, we found that a combination of Huang qi (*Radix Astragali seu Hedysari*) and Da huang (*Radix et Rhizoma Rhei*), with tonifying and purging effects, respectively, appeared in a lot of TCM formulas and was reported to be effective for the diseases coexisting deiciency and excess syndromes such as CRF, which is consistent with TCM's basic treatment principles [2–4].

In this study, CRF model rats were produced by adenine. Drugs chosen for intervention were Astragalus polysaccharide (APS), the most intensively observed and effective monomer from Huang qi (*Radix Astragali seu Hedysari*), and Rhein, an anthraquinone from Da huang (*Radix et Rhizoma Rhei*), which has been patented for CRF treatment. The effects of APS and Rhein combination on renal function, renal pathology, and tubular cell apoptosis in model rats were assessed to investigate the protective mechanism of APS and Rhein on the kidney.

## 2. Materials and Methods

### 2.1. Experimental Materials

Thirty-seven, clean-grade, male Wistar rats, weighing 150–180 g were provided by the Experimental Animal Center of Southern Medical University (license number: SCXK [Guangdong] 2006-0015). The rats were reared in natural light conditions at a humidity of 50–60% and a constant temperature of 20 ± 3°C. Before the experiment, all rats were housed with food and water, freely available for 1 week, so they could adapt to the environment. Adenine (dissolved in normal saline before use) was purchased from Amresco Inc (Solon, OH, USA). APS (purity, 70%; freshly dissolved in 0.5% sodium carboxymethylcellulose [CMCNa] in saline before use) and Rhein (purity, 98%; freshly dissolved in CMCNa before use) were purchased from Nanjing Zelang Medical Technology Co., Ltd. (Nanjing, China). A hematoxylin and eosin (HE) staining kit was purchased from Solarbio (Beijing, China). A terminal deoxynucleotidyl transferase dUTP nick end labeling (TUNEL) apoptosis kit was purchased from Dingguo Changsheng Biotechnology Co., Ltd. (Beijing, China).

### 2.2. Animal Grouping, Modeling, and Administration

The 37 male Wistar rats were randomly divided into a control group (*n* = 7), a model group (*n* = 10), a low-dose APS (200 mg/kg/d) and Rhein (25 mg/kg/d) combination group (*n* = 10), and a high-dose APS (400 mg/kg/d) and Rhein (100 mg/kg/d) combination group (*n* = 10). The control group received normal food and water during the experiment. In the other 3 groups, a 2% adenine suspension was given at 200 mg/kg/d by gavage every morning to induce CRF. Every afternoon, the low- and high-dose APS plus Rhein groups were administered APS and Rhein by gavage at their respective doses. The rats in the model group were given an equal volume of distilled water by gavage. On the 28th day of the experiment, 6 rats were randomly selected from each group and underwent tail cutting, and a blood sample was collected from each rat. Serum creatinine (SCr) and blood urea nitrogen (BUN) levels were measured to determine whether the animal model was successfully established. During the subsequent 3 weeks, the model group and the low- and high-dose APS plus Rhein groups were given adenine by gavage every other day, and the other procedures remained the same. In total, the treatment period was 7 weeks.

### 2.3. Specimen Collection

Before the operation, food and water were not given to the rats for 12 h and 4 h, respectively. After giving anesthesia via an intraperitoneal injection of sodium pentobarbital (40 mg/kg), we collected a 5 mL blood sample from the abdominal aorta of each rat without anticoagulant. After standing for 20 min, the blood samples were centrifuged for 20 min at 2000 rpm. Then, the separated serum was stored in Eppendorf tubes for later measurement of kidney function and electrolytes. Meanwhile, the kidneys were quickly removed, decapsulated, washed with saline at 4°C, and weighed. One-fourth of the left kidney was fixed with 4% paraformaldehyde and stained with HE. The right kidney and three-fourths of the left kidney were stored at −80°C for later use.

### 2.4. Rental Pathological Changes

The rat kidneys' color and morphology were assessed by visual inspection with the unassisted eye. Rat kidney injury was evaluated using HE staining, and pathological scoring was performed as follows: tissues were embedded in paraffin, cut into 2 *μ*m slices, dewaxed with water, stained with hematoxylin for 10 min, differentiated with 0.5% hydrochloric acid alcohol for 20 s, and stained with eosin for 5–10 min, after which they were then dehydrated, cleared, mounted, and photographed by a high-power microscope.

### 2.5. Examination of Tubular Cell Apoptosis by TUNEL

Paraffin sections of kidney tissue (4 *μ*m) were dewaxed, digested with proteinase K, washed with PBS, incubated with blockers in the dark at room temperature, and then incubated with the TUNEL reaction mixture in the dark at 37°C. Blocking buffer was added after the reaction was terminated, followed by incubation with Streptavidin-HRP at 37°C. Diaminobenzidine was then added for color development, and hematoxylin was added for counterstaining. Finally, the sections were dehydrated, cleared, and mounted. TUNEL-positive nuclei that stained brown were observed under an optical microscope at a 200-fold magnification. Six slices were randomly selected from each group. Apoptotic cells were counted within 3 fields of view. The total number of apoptotic cells was then calculated for each group.

### 2.6. Detection of IRE1, CHOP Protein Expression by Western Blot

The renal tissues were treated with RIPA buffer on ice to lyse for 15 minutes and were centrifuged at 4°C, 12000 rpm for 15 min. Then the supernatant was transferred to a fresh tube and all the samples were stored at −20°C. After the sample concentrations were estimated by BCA assay, they were added 1/4 vol of 5x SDS loading buffer, and heated in boiling water. 5 min later, they were loaded into precast 1 mm, 12-well gel and run at 120 V for approximately 1 hour. After running, SDS-PAGE gels were transferred to PVDF membranes, which was pretreated in methanol and transfer buffer, at 100 V for 90 minutes. Then the membranes were soaked in 5% skim milk for blocking for 2 hours at room temperature with two washes of TBST and incubated with primary antibodies overnight at 4°C. After three washes of TBST, the membranes were incubated with secondary antibody at room temperature for 1 hour and washed with TBST for another 3 times. Subsequently, the membranes were developed by ECL method and the developed films were analyzed using Quantity One software.

### 2.7. Statistical Analysis

All data were processed with SPSS 17.0 (SPSS, Chicago, IL, USA). Measurement data were presented as mean ± standard deviation (mean ± SD). Comparison among groups was conducted using a one-way analysis of variance (ANOVA). If homogeneity of variance was present, a Student-Newman-Keuls (SNK) test was used; otherwise, a Tamhane's test was performed.

## 3. Results

### 3.1. General Conditions of Rats

In the control group, the rats had thick and shiny hair, active behavior, a normal food and water intake, blue-black and well-formed stools, and normal urination during the experiment.

The rats in the model group began to take less food and water and had formed slightly loose, stools the day after modeling. As of day 8 after modeling, the rats had darkened, thinning hair and a loss of hair; had a worse mental state; exhibited the phenomenon of clustering, arching of the back, curling, and slow response; had less food and water intake; and had loose and less stools. From day 30 after modeling, 4 rats died intermittently. The deaths might have been caused by the modeling, adverse reactions to the monomer, individual differences, or experimental operations.

In the low-dose APS plus Rhein group, since the day after modeling, the rats ingested less food and water and had slightly increased stools; stools were formed but loose, smelly, and reddish, which was probably the color of the APS metabolites. From day 9, rats began to participate in slightly fewer activities and had a slightly worse mental state; however, their stools were the same as before. From day 15, the rats had dull, pale yellow, thin hair and a loss of hair; had a poor mental state; and exhibited the phenomenon of clustering, curling, and arching of the back; and had similar food and water intake and stools as before the experiment. Two rats died during the experiment that might have been caused by the experimental operation, modeling, or adverse reactions to the monomer.

In the high-dose APS plus Rhein group, since the day after modeling, the rats had less food and water intake; their stools had increased in amount and became loose, smelly, and a brownish-reddish color; their stools sometimes were in the shape of a string of beads; and the anus of the rats was often dirty. Compared with the control group, the hair color and mental state were slightly worse, but no other differences existed. Two rats died during the experiment that was probably caused by mistakes in the experiment operation.

In summary, the high-dose treatment group was better than the model group with respect to general condition, and great differences were observed in stool volume and shape between the 2 groups.

### 3.2. Comparisons of Kidney Weight and Index

The kidney weights of the treatment groups and model group were significantly higher than that of the control group (*P* < 0.01). The kidney weight of the model group was significantly higher than that of the treatment group (*P* < 0.01). No significant differences were observed between the 2 treatment groups (*P* > 0.05).

Meanwhile, the kidney index (which was calculated as follows: kidney index = kidney weight/body weight × 10^3^) of the model group was significantly increased, as compared with that of the control group (*P* < 0.05). Furthermore, the kidney indexes of the treatment groups were significantly increased, as compared with that of the model group (*P* < 0.05) (Table 1).

### 3.3. Effects of the APS and Rhein Combination on BUN, SCr, Calcium, and Phosphorus Concentrations

The BUN levels were in the normal range in the control group, while they were increased in model group; moreover, significant differences were observed between the 2 groups (*P* < 0.01). In addition, the BUN levels were significantly decreased in the treatment groups, as compared with that of the model group (*P* < 0.01).

As for the SCr levels, they were in the normal range in the control group, but they were increased in the model group; significant differences were observed between the 2 groups (*P* < 0.01). The SCr levels were significantly decreased in the treatment groups, as compared with that of the model group (*P* < 0.01).

The serum calcium and phosphorus concentrations were in the normal range in the control group. However, in the model group, the serum calcium concentration was decreased, and the phosphorus concentration was increased; significant differences were observed in both serum calcium and phosphorus concentrations between the control and model groups (both *P* < 0.01). Furthermore, the serum calcium concentration in the high-dose group was significantly higher than that of model group (*P* < 0.01), while there was no significant difference demonstrated in the control group (*P* > 0.05). The serum phosphorus concentration in the treatment groups was significantly decreased, as compared with that of the model group (*P* < 0.01) (Table 2).

### 3.4. Renal Pathological Changes

We visually inspected the rats' kidneys. In the control group, the rats' kidneys were reddish-brown and difficult to decapsulate; the kidneys had a smooth surface without spots, were of relatively small size, and had a clear boundary between the cortex and medulla on the frontal section. As for the model and low-dose groups, the rats' kidneys were pale and easy to decapsulate and had dark spots on the surface, were of the largest size, and had an unclear boundary between the cortex and medulla on the frontal section. Meanwhile, in the high-dose group, the rats' kidneys were between a reddish-brown and white color, and the kidneys were easy to decapsulate, had dark spots on the surface, and had a relatively clear boundary between the cortex and medulla on the frontal section. Moreover, the kidney size of the rats in the high-dose group was larger than that of control group but smaller than that of model and low-dose groups.

We also assessed the HE staining results as follows.

**Figure 1:**
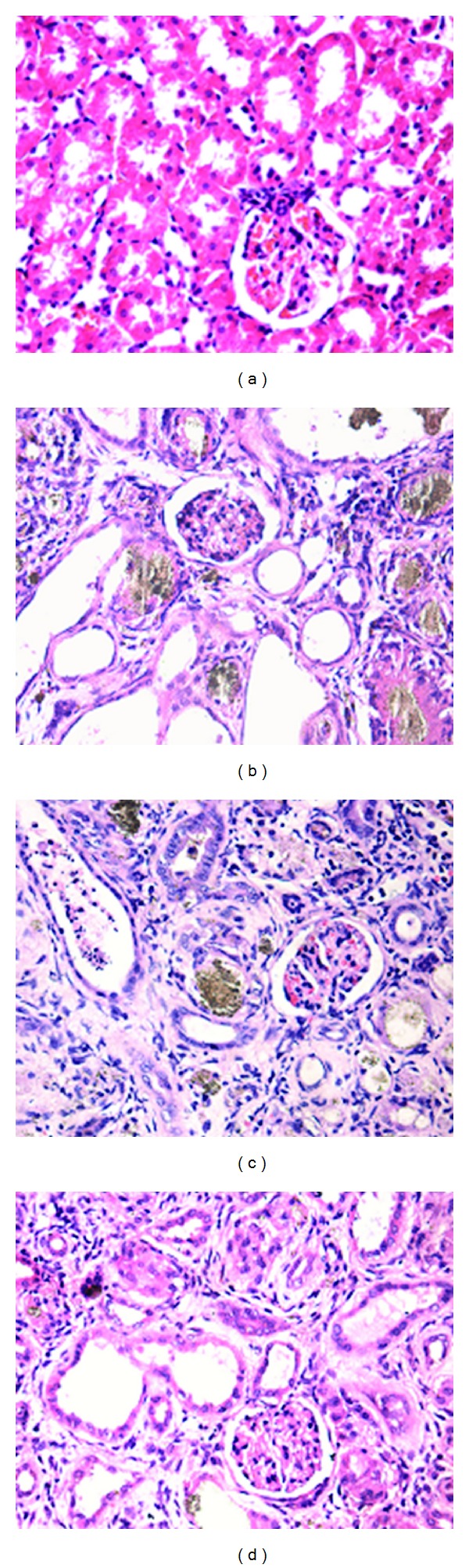
Pathological pictures with HE staining (×200). (a) The control group had no significant pathological changes. (b) The model group showed contracted glomeruli, distended glomerular capsules, and heavy deposition of adenine metabolites in the lumen. Interstitial inflammatory cell and fibroblast infiltration as well as interstitial deposition of adenine metabolites were also observed. (c) The low-dose group demonstrated the same pathological changes as the model group but had a reduced deposition of adenine in the interstitium and lumen. (d) The high-dose group appeared slightly contracted glomeruli, relatively distended glomerular capsules without adenine deposition, a small amount of adenine deposition in the tubules, and little interstitial inflammatory cells and fibroblast infiltration.

- The control group had no significant hypertrophy, contracted or distended glomerular capsule, basement membrane hyperplasia, or inflammatory cell infiltration within the glomeruli. Juxtaglomerular tubules and medullary tubules were tightly packed and in a regular shape. There was only a small amount of interstitium. Hypertrophy of the juxtaglomerular apparatus was observed, but it was not common and had no pathological significance.
- The model group was observed to have contracted glomeruli and distended glomerular capsules without deposition of adenine metabolites along with atrophy of the proximal tubule, distal tubule, medullary loop, and collecting duct; and stenosis or dilatation of the lumen or even disappearance of the tubules. The epithelial cells had a reduced size and a heavily stained nucleus. A heavy deposition of adenine metabolites was found in the lumen. Interstitial inflammatory cells and fibroblast infiltration, as well as interstitial deposition of adenine metabolites, were also observed.
- The low-dose group demonstrated the same pathological changes as the model group but had a reduced deposition of adenine in the interstitium and lumen.
- In the high-dose group, the glomeruli were slightly contracted, and the glomerular capsules were relatively distended and had no adenine deposition. The tubules were of a regular shape, with a small amount of adenine deposition. Interstitial inflammatory cells and fibroblast infiltration were at a level lower than that of the model and low-dose groups (Figure 1). The HE staining results of this experiment revealed that the glomerular injury was mild and that major malformations were tubular stenosis or dilatation and interstitial cell proliferation and invasion.

### 3.5. Effects of the APS and Rhein Combination on Tubular Cell Apoptosis

**Figure 2:**
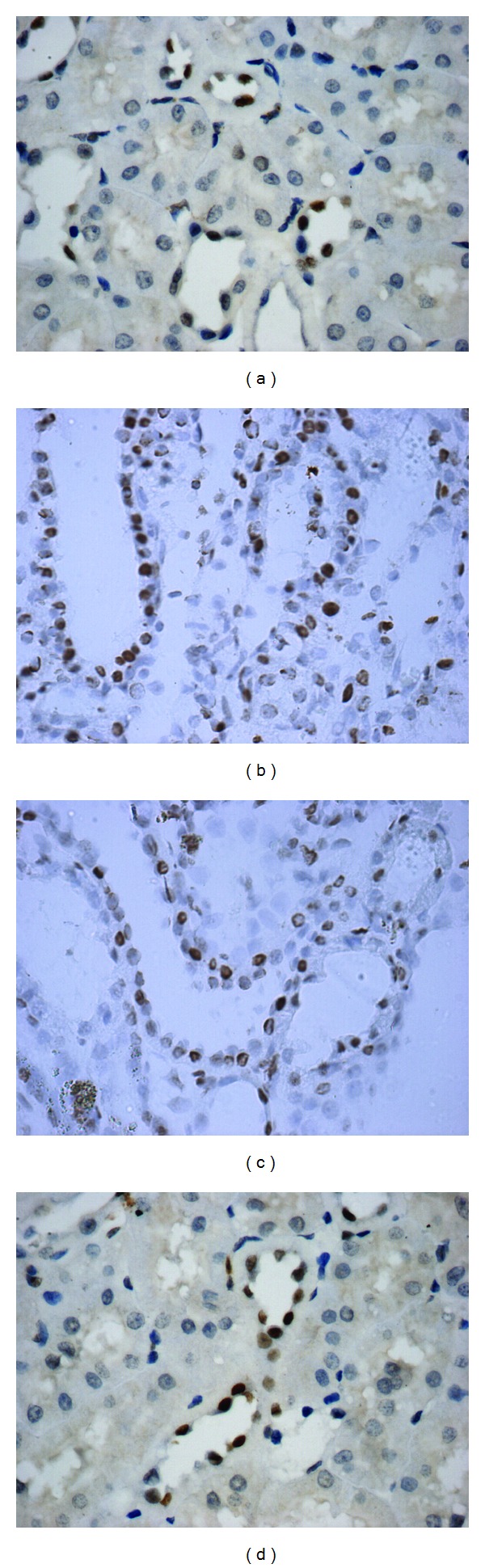
Effects on tubular cell apoptosis by TUNEL (×400). Mainly the tubular cells and rarely the glomerular cells were apoptotic in each group. (a) The control group had a small number of apoptotic tubular cells. (b, c) The model and low-dose groups showed a large number of apoptotic tubular cells. (d) A relatively small number of apoptotic tubular cells were in the high-dose group.

The TUNEL results revealed that mainly the tubular cells and rarely the glomerular cells were apoptotic in each group. A small number of apoptotic tubular cells were detected in the control group. The largest number of apoptotic tubular cells was observed in the model and low-dose groups. Additionally, a relatively small number of apoptotic tubular cells were in the high-dose group. Pairwise comparisons between each group were statistically significant (*P* < 0.01) (Table 3 and Figure 2).

### 3.6. Effects of the APS and Rhein Combination on IRE1, CHOP Protein Expression

**Figure 3:**
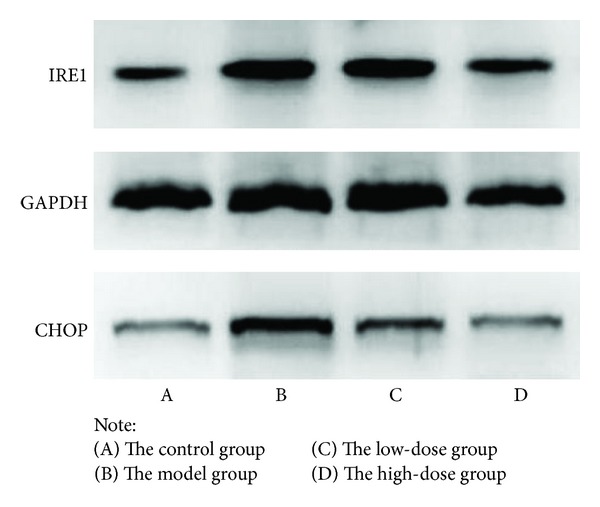
Effects on IRE1, CHOP protein expression.

The normal group had lower protein expressions of IRE1 and CHOP than that of the model group (*P* < 0.05). Compared with the model group, the protein expressions of IRE1 in the treatment groups were decreased, while there were no significant differences between the treatment groups and the normal group. As for CHOP, the protein expression in the high-dose treatment group increased compared with the normal group (*P* < 0.05), while there were no significant differences between the low-dose treatment group and the normal group (shown in Table 4 and Figure 3).

## 4. Discussion

TCM pathogenesis of CRF is deficiency in origin and excess in superficiality. Deficiency in origin refers to spleen and kidney deficiency, while excess in superficiality refers to stasis of toxic dampness. Therefore, the principle of treating CRF is reinforcement and elimination in combination. The combination of Huang qi (*Radix Astragali seu Hedysari*) and Da huang (*Radix et Rhizoma Rhei*) is common for treating CRF in Chinese traditional medicinal formulas, such as the Qize Decoction [5], Shenqi Jiedu Decoction [2], and Uremic Clearance Granule. Astragalus, with sweet flavor and tepid property has the function of tonifying deficiency of lung, spleen, and kidney and inducing diuresis to alleviate edema; Rhubarb, with bitter flavor and cold property, has the function of discharging stagnated heat and toxic dampness. These two drugs are matched to tonify deficiency and purge excess, so as to strengthen spleen and kidney to remove dampness, resolve turbidity, and expel toxins.

Modern medicine thinks that the pathogenesis of CRF includes intact nephron hypothesis, trade-off hypothesis, glomerular hyperfiltration hypothesis, inflammatory mediators, and cytokines hypothesis. The main pathologic changes of CRF are glomerular sclerosis, tubular atrophy, and interstitial fibrosis. APS and Rhein are the main active ingredients of Huang qi and Da huang, respectively. It has been reported that APS can significantly reduce kidney weight/body weight of the diabetic nephropathy rats, improve renal function, and retard the development of glomerular sclerosis [6] and relieve inflammatory reaction in rats with acute pyelonephritis [7]. Rhein can decrease renal interstitial fibrosis in mice with unilateral ureteral occlusion by inhibiting the activity of fibroblasts [8]; suppresses high glucose- and angiotensin II-induced hypertrophy of the renal proximal tubular epithelial cells [9]; decreases lipid levels; and reduces urinary albumin excretion and extracellular matrix in diabetic mice [10].

In the research, CRF model rats induced by adenine showed obvious impairment of glomerular filtration function and disturbance of calcium and phosphorus metabolism, and renal pathology was also consistent with clinical pathology of CRF patients. The APS and Rhein combination could alleviate the functional damage of glomeruli, relieve pathologic changes of tubules, and decrease proliferation and invasion of the interstitial inflammatory cells.

Clinical researches showed that cell apoptosis widely appeared in CRF patients and inhibition of apoptosis could delay the progress of CRF disease and reduce or prevent occurrence of the related complications [11]. Apoptosis, which is triggered by internal or external causes, is a natural process of programmed cell death. Apoptosis occurs normally as a homeostatic mechanism to maintain cell populations or as a defense manner against harmful stimuli, whereas excessive apoptosis could result in various diseases. So far, the mechanism of cell apoptosis is still obscure. It is commonly accepted that there are three apoptotic pathways: mitochondrial pathway, death receptor pathway, and endoplasmic reticulum pathway. And endoplasmic reticulum stress (ERS) is the core content of endoplasmic reticulum pathway. ERS arises in various conditions or circumstances such as hypoxia, oxidative injury, high-fat diet, hypoglycemia, and viral infection, which disturbs the homeostasis of the ER. Multiple disturbances can cause accumulation of unfolded proteins in the ER, which results in the unfolded protein response (UPR). UPR is regulated by a set of transmembrane proteins, including inositol-requiring enzyme 1 (IRE1) [12]. When the unfolded protein exceeds a threshold, UPR triggers downstream signaling molecules (CHOP, caspase family, etc.) [13] and leads to cell apoptosis.

This research proved that the expression level of apoptosis in the CRF model rats was significantly higher than that of the other groups, and APS and Rhein combination could effectively inhibit the apoptosis of renal tubular cells in the rats. Our previous researches showed that excessive ERS occurred in the renal tissues of the CRF model rats induce by adenine on the basis of significantly increased the expression of Caspase-12 in the rats [5]. This research further found that the protein expressions of IRE1 and CHOP increased significantly in the CRF model rats by Western blot, while APS and Rhein combination could reduce the protein expressions of IRE1 and CHOP to alleviate ERS reaction in the rats. So we proposed that APS and Rhein combination could inhibit the apoptosis of renal tubular cells in animal models, improve renal function, whose mechanism might be related to inhibition of excessive expression of ERS-related molecules, and keeping homeostasis of intracellular environment.

## Figures and Tables

**Table 1:** Comparisons of kidney weight and index among groups (mean ± SD).

Group	Kidney weight (mg)	Kidney index (×10^3^)
Control group (*n* = 7)	1.69 ± 0.15	5.52 ± 0.25
Model group (*n* = 6)	5.33 ± 0.54^a^	29.36 ± 2.48^a^
Low-dose group (*n* = 8)	3.66 ± 0.44^b^	20.07 ± 3.65^b^
High-dose group (*n* = 8)	3.64 ± 0.45^b^	16.83 ± 2.30^b^

Notes: ^a^ *P* < 0.01 compared with control group; ^b^ *P* < 0.01 compared with model group.

**Table 2:** Comparisons of BUN, SCr, Ca, and P among groups (mean ± SD).

Group	BUN (µmol/L)	Scr (mmol/L)	Ca (mmol/L)	P (mmol/L)
Control group (*n* = 7)	5.93 ± 0.50	30.29 ± 3.35	2.25 ± 0.05	2.04 ± 0.13
Model group (*n* = 6)	64.83 ± 10.08^a^	324.67 ± 51.93^a^	1.77 ± 0.20^a^	5.18 ± 0.24^a^
Low-dose group (*n* = 8)	32.93 ± 8.40^c^	181.50 ± 55.25^c^	1.78 ± 0.30	3.82 ± 0.92^c^
High-dose group (*n* = 8)	21.09 ± 5.82^c^	100.88 ± 45.65^c^	2.20 ± 0.41^b^	3.00 ± 0.66^c^

Notes: ^a^ *P* < 0.01 compared with control group; ^b^ *P* < 0.05 compared with model group; ^c^ *P* < 0.01 compared with model group.

**Table 3:** Comparison of the number of apoptotic cells among groups (mean ± SD).

Group	Number of apoptotic cells
Control group (*n* = 6)	18.67 ± 3.78
Model group (*n* = 6)	75.67 ± 9.44^a^
Low-dose group (*n* = 6)	49.67 ± 3.40^b^
High-dose group (*n* = 6)	28.83 ± 2.46^c^

Notes: ^a^ *P* < 0.01 compared with control group; ^b^ *P* < 0.05 compared with model group; ^c^ *P* < 0.01 compared with model group.

**Table 4:** Comparisons of IRE1, CHOP protein expression among groups (mean ± SD).

Group	IRE1	CHOP
Control group (*n* = 7)	0.05 ± 0.01^b^	0.04 ± 0.01^b^
Model group (*n* = 6)	0.17 ± 0.04^a^	0.13 ± 0.02^a^
Low-dose group (*n* = 8)	0.11 ± 0.03^b^	0.10 ± 0.02^a^
High-dose group (*n* = 8)	0.06 ± 0.02^b^	0.05 ± 0.01^b^

Notes: ^a^ *P* < 0.05 compared with control group; ^b^ *P* < 0.05 compared with model group.
